# Navigating the Legislative Interventions, Challenges, and Opportunities in Revolutionizing Textile Upcycling/Recycling Processes for a Circular Economy

**DOI:** 10.1021/acssusresmgt.4c00242

**Published:** 2024-10-11

**Authors:** Maria Saif, Rubén Blay-Roger, Muhammad Zeeshan, Luis F. Bobadilla, Tomás Ramírez Reina, Muhammad Asif Nawaz, José Antonio Odriozola

**Affiliations:** † Department of Inorganic Chemistry and Materials Sciences Institute of Seville (ICMS), University of Seville-CSIC, Seville 41092, Spain; ‡ School of Computing and Engineering, University of South Carolina, Columbia, South Carolina 29208, United States

**Keywords:** Circular Economy, Textile Recycling, Waste Management, Resource Recovery, Sustainable Fashion

## Abstract

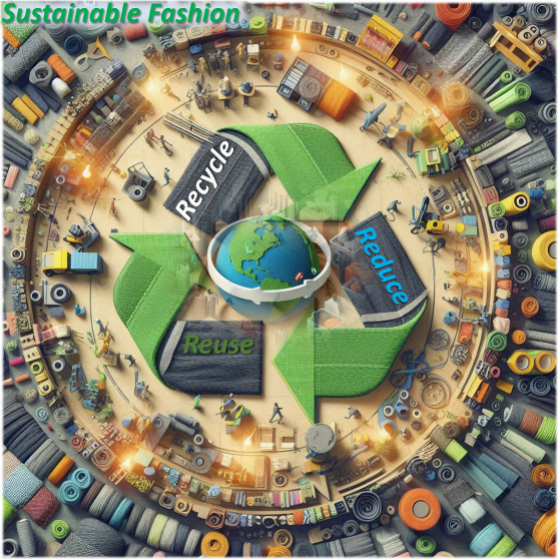

Embracing a circular economy in the textile industry represents a crucial step toward sustainability, where fashion and textile sectors contribute significantly to CO_2_ emissions. However, transitioning from a linear “take-make-waste” model to circularity, poses multifaceted challenges, that highlight the staggering volume of annual textile waste surpassing industry predictions, thus emphasizing the urgent need for comprehensive strategies. Despite advancements in recycling technologies, challenges persist in collecting and sorting textile waste, where fragmentation in waste management and recycling processes hinders effective management of post-consumer waste. Addressing these challenges demands elevated efforts in collection, sorting, and pre-processing, alongside regulatory interventions to drive enhanced waste collection and circular business models. Efforts are underway to promote sustainable textile recycling, with initiatives like the EU’s Sustainable and Circular Textiles Strategy aiming to reduce reliance on virgin resources. However, achieving a circular textile market in the near future requires collaborative action and innovative solutions. Though challenges in scaling and technological limitations still remain, recent breakthroughs in textile-recycling technologies offer promise, signaling a shift toward scalable and sustainable alternatives to virgin fibers, where bio-based chemical processes, and thermochemical recycling processes present transformative opportunities. Where, bold scaling targets, collaborative efforts, and short-term funding support narrated in this perspective article are imperative to accelerate the transition to a circular textile economy, thus delving into the pivotal role of textile recycling, tracing the evolution of recycling technologies, and addressing critical challenges hindering widespread adoption.

## Abstract 2

This study emphasizes the efficient textile waste management, innovative recycling technologies, and legislative frameworks to promote environmental sustainability and resource conservation in the textile industry for circular economy.

## Introduction to the Sustainable Fashion by Upcycling and Recycling

**Figure 1:**
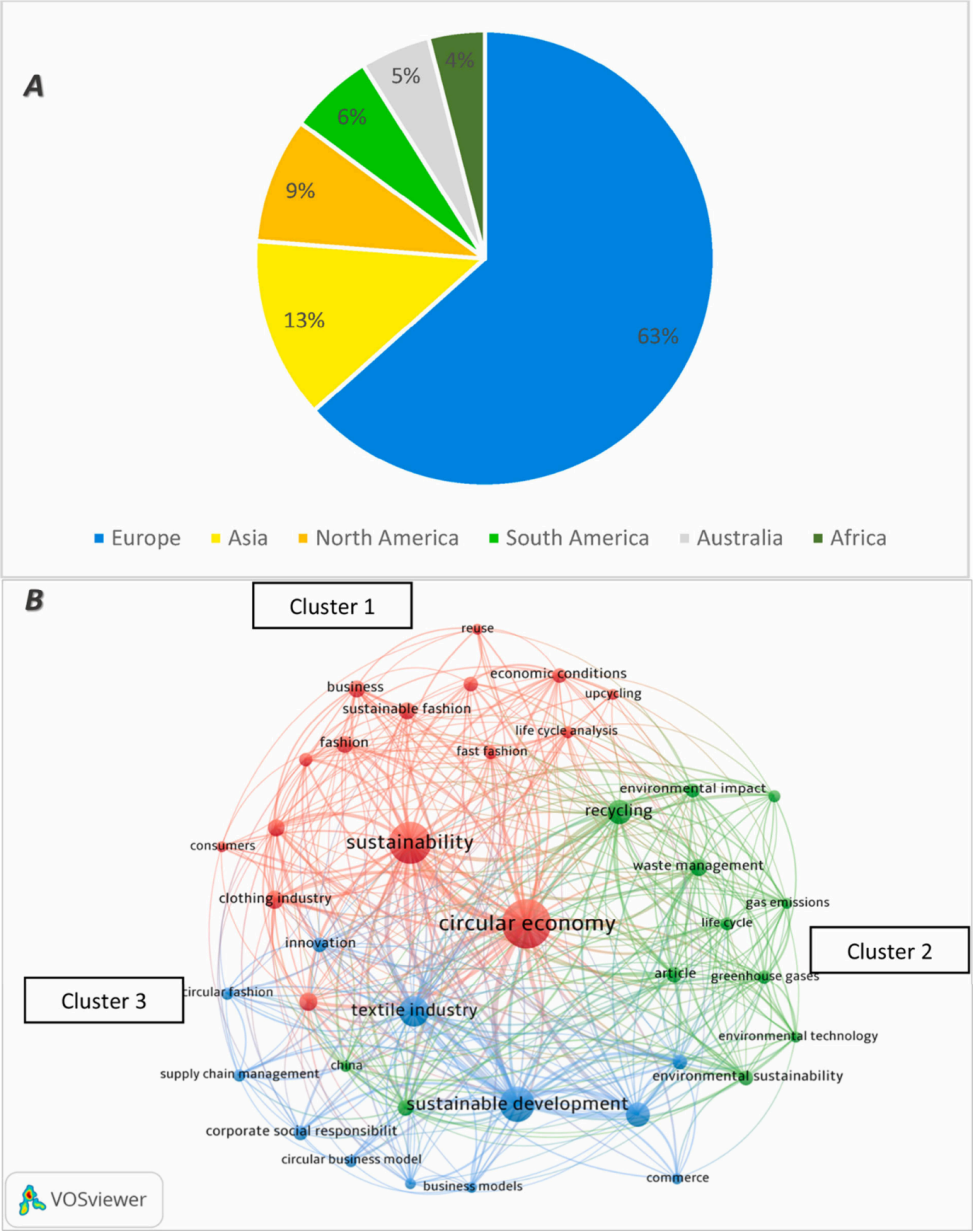
(A) Ratio of the carbon footprint by textile sector on different regions. Reproduced from ref (20). Copyright 2023 Elsevier. (B) Circular economy and sustainability goals closely crosslinked with textile recycling and post-consumer behavior derived from recent studies. Reproduced from the ref (2). Copyright 2024 Elsevier.

The global fashion and textile industries find themselves at a critical juncture, where the shift toward circularity in textile recycling presents a defining moment, as these industries, long criticized for their environmental impact, significantly contribute to global CO_2_ emissions (Figure 1A).^1−3^ Where, shifting away from the linear “take-make-waste” model demands a comprehensive strategy, especially focusing on upstream operations like material production.^4,5^ Closed-loop, fiber-to-fiber recycling holds enormous potential for revolutionizing sustainability practices within the industry.^6^ However, realizing this vision requires overcoming critical challenges in technology advancement, scaling capacity, and fostering collaboration across the value chain.^7,8^ This urgency is emphasized by the close link between circular economy and sustainability goals with textile recycling and post-consumer behavior (Figure 1B).^2,9^ Fast fashion, characterized by the frequent introduction of new styles at low prices, has led to a significant increase in clothing production and waste. In Europe alone, 7–7.5 million tons of textile waste are generated annually, projected to increase to 8.5–9 million tons by 2030.^10^ This waste, primarily from discarded clothing and household textiles, poses grave threats to ecosystems globally as the waste effluent is mainly composed of multiple hazardous and refractory contaminants (acids, alkalis, dyes, toxic elements, and diversiform organic compounds).^11^ Efforts to address this challenge are hampered by fragmentation in waste management and recycling processes. Presently, only 30–35% of post-consumer household textile waste is collected, with approximately 1– 2% undergoing fiber-to-fiber recycling due to technological limitations in processing mixed and degraded fibers.^12−15^ To achieve a circular textiles market, elevated efforts in collection, sorting, and pre-processing are essential, alongside regulatory initiatives to drive enhanced waste collection and circular business models,^16^ where the Sustainable and Circular Textiles Strategy would aim to reduce reliance on virgin resources, prioritizing durable, recyclable products crafted from recycled fibers.^17^ As we journey toward a circular economy, managing textile waste emerges as a critical consideration for global fashion and textile industries.^18^ With waste volumes projected to surge, driven by consumption and population growth, the maturation of recycling technologies necessitates a consistent supply of well-sorted feedstocks. EU regulations mandating separate textile waste collection by 2025, alongside the influence of producer responsibility organizations (PROs), hold the potential to significantly boost collection rates for post-consumer household waste to 50 % by 2030.^19^

Despite progress, achieving comprehensive collection of all textile waste will require concerted efforts in view of a possible surge (by triple or quadruple) in the volume of textile waste available for recycling, driven by factors like increased collection rates and reduced exportation. Textile recycling offers a dual advantage of reducing resource depletion and energy-intensive processes by substituting recycled materials for virgin ones, while also addressing the mounting issue of textile waste accumulation, providing a sustainable alternative to landfill disposal.^21^ Recent breakthroughs in textile-recycling technologies signal a shift toward scalable and sustainable alternatives to virgin fibers, underlining the industry’s commitment to sustainability. Processes like chemical recycling, mechanical recycling, and upcycling have emerged as powerful solutions to combat the environmental impact of the fashion industry.^22^ Sustainable thermochemical recycling presents another avenue for revolutionizing textile recycling along with the closed-loop systems through bio-based chemical processes like hydrolysis and fermentation.^23,24^ While, embracing enzymatic and biological processes in this concern unlocks the potential for sustainable textile recycling, harnessing nature’s power to transform end-of-life textiles into valuable resources.^25,26^ Yet, unlocking its full benefits demands collective action from industry stakeholders, policymakers, and researchers.^18^ Addressing structural and technical hurdles in collection, sorting, and preprocessing is crucial for scaling global textile recycling efforts successfully.^13^ Regulatory measures, such as prohibiting the export of unsorted textile waste, could encourage consolidation in the textile sorting sector, simplify feedstock sourcing for recyclers, and promote a more sustainable textile recycling ecosystem. Therefore, to combat the environmental repercussions of fast fashion, efforts need to be committed to minimizing textile waste and enhancing the lifecycle and recycling of textiles. The guide in this Perspective provides insights into various recycling methods like mechanical, hydrothermal, biological, chemical, and physical recycling, along with examples of innovative recycling systems. It also offers recommendations for fiber selection, fabric processing, design and construction, labeling, and closing the recycling loop, aiming to facilitate the recycling process and promote a sustainable fashion industry.

## Confronting the Collecting and Sorting Dilemmas for Textile Upcycling and Recycling

**Figure 2:**
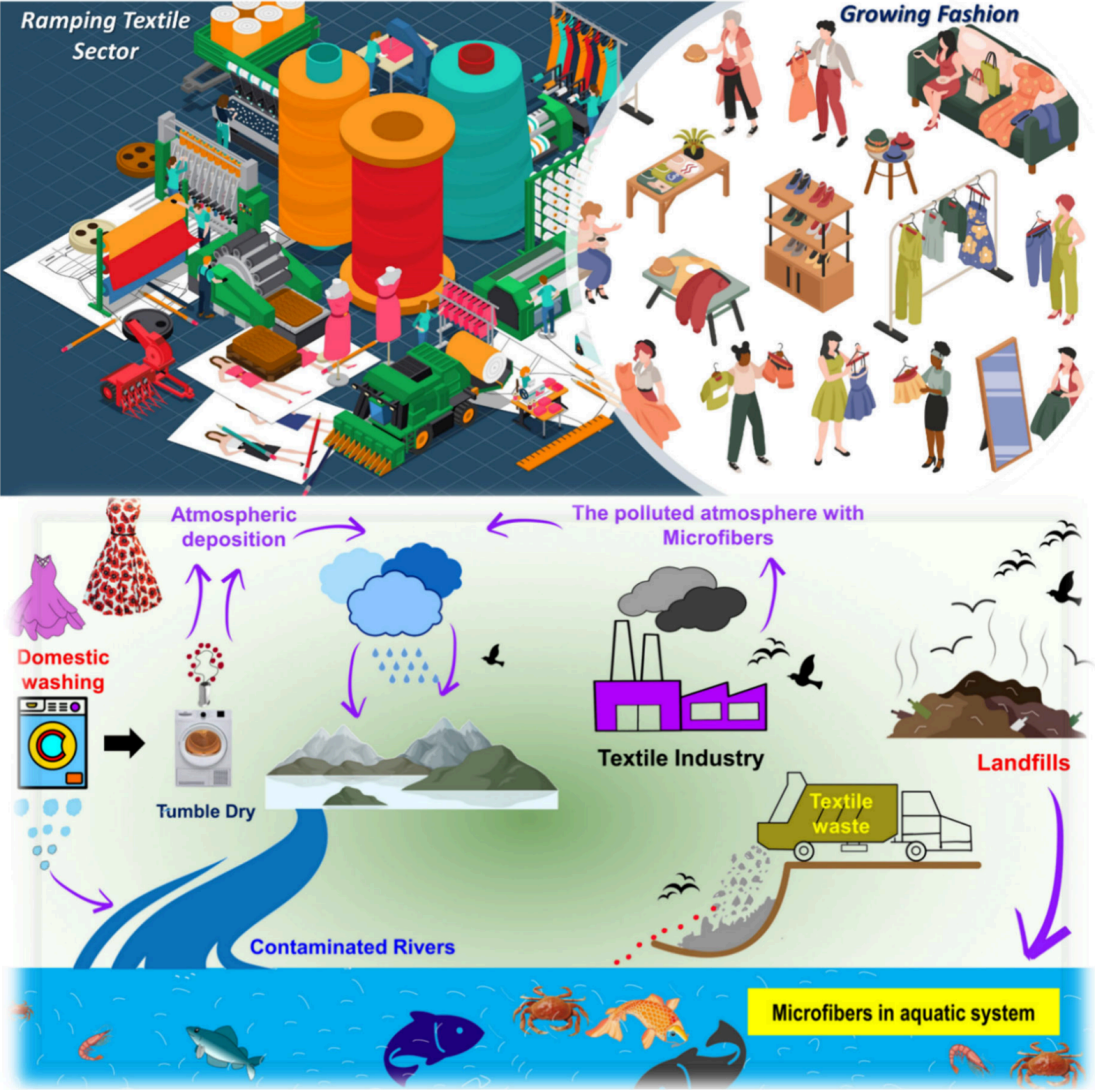
(A–C) The impact of ramping textile sector with the growing fast fashion on the environment. (C) Reproduced from ref (29). Copyright 2023 MDPI.

Various strategies need to be implemented, including eco-design requirements for textiles and extended producer responsibility schemes, proposing stricter measures to curb excessive textile production and consumption, with an emphasis on human rights, environmental protection, and animal welfare. While progress has been made in recycling technologies, addressing challenges related to feedstock availability remains crucial. Within these challenges lies a transformative opportunity: upcycling. By conversion of textile waste into new fibers for manufacturing, upcycling offers a scalable and sustainable solution to tackle the root cause of the waste problem while creating fresh economic prospects. Despite its promise, upcycling faces barriers to scaling like recycling, including challenges in collection, sorting, and preprocessing as well as technological limitations in handling fiber blends and high preprocessing costs. However, to realize the full potential of upcycling, concerted efforts and significant investments are estimated across the textile recycling value chain. In the pursuit of sustainable solutions, the textile industry, driven by economic progress and rising living standards, encounters significant environmental challenges and formidable obstacles, particularly in terms of feedstock availability for recycling/upcycling.^27^ The seamless operation of recycling technologies relies heavily on a steady stream of textile waste, yet challenges persist in its collection, sorting, and pre-processing, post-consumer waste, offering potential for expanding recycled yarn and fabric production and potentially displacing raw materials.^12^ The industry’s fast fashion business model, marked by mass manufacturing and affordability, has led to a surge in waste generation (as shown by Figure 2), illustrating the environmental impact of textile consumption and ramping textile sector with the growing fast fashion, including landfill water pollution and greenhouse gas emissions (estimated to account for 10% of global carbon emissions).^1,28,29^ Usually, textile waste manifests in various forms, including solid waste, liquid waste, and gas waste. Solid waste encompasses everything from scraps and damaged materials to leftover fabrics and packaging materials. Liquid waste results from the chemical-intensive processes involved in textile manufacturing, contributing to water pollution due to its high toxicity. Gas waste, generated during processing and fueled by boilers, adds to air pollution, further straining environmental resources. Disposing of textile waste, such as the short fibers known as willow waste, presents challenges, as they are unsuitable for textile applications and often end up in landfills. Proper treatment of such waste is crucial to mitigate its environmental impact. The sheer ubiquity of textiles in various products underscores the urgency of addressing the textile industry’s waste problem. Textiles are intricate amalgamations of buttons, zippers, coatings, prints, and impurities, making their processing a Herculean task. Fabric blends, especially those combining multiple materials, further complicate matters. Traditional mechanical separation proves insufficient as these fibers are intricately intertwined, hindering efficient recycling. Of these blends, blended textiles, particularly polyester–cotton (polycotton) blends, are noted as particularly challenging, dominating the market with their complexity and making efficient recycling difficult.^24,30^

Tackling the technological hurdles in recycling such blends is paramount. Once breakthroughs are made in separating blended textile waste, a transformative shift awaits. Textile wastes, once destined for landfills or incineration, can now be reincarnated, maximizing material circulation and reducing reliance on virgin resources. This marks the final piece in the puzzle of achieving a zero-waste textile economy, ushering in an era where environmental impacts are minimized and sustainability reigns supreme. Recycling the diverse textiles demands meticulous sorting and processing to avoid fiber degradation and ensure closed-loop recycling’s effectiveness. The complex nature of textile polymers, poses significant challenges for recycling,^31^ where compounding the issue are synthetic dyes and pigments used in fabric coloring, which hinder chemical recycling and necessitate thorough cleansing for mechanical recycling.^8,32^ The textile sector’s diverse landscape, spanning manufacturers, retailers, and consumers, poses another logistical puzzle when it comes to collecting waste.^33^ Coordinating this fragmented supply chain requires strategic coordination and outreach. Additionally, changing consumer behavior to encourage recycling participation is crucial yet complex, as many still opt for landfill disposal despite growing awareness.^12,34^ Moreover, ensuring the quality of collected waste remains paramount, as contamination from mixed materials or chemical residues can impede upcycling efforts. The sheer diversity of textile waste, ranging from clothing to industrial scraps, presents a formidable sorting challenge. Advanced technology is essential to efficiently categorize materials made from various fibers like cotton, polyester, and nylon.^35^ Accurate sorting based on fiber characteristics is essential, yet clothing labels often lack detailed fiber composition information, complicating the task. Despite technological advancements, reliance on manual sorting persists in many facilities, leading to inefficiencies and classification errors, which direly needs to automate the process, striving to generate the high-quality fractions pivotal in surmounting the recycling conundrum.^36^ While near-infrared technology (NIR) show promise, their garment separation system remains unvalidated, with few alternative solutions being available. Innovations in waste collection, sorting technologies, and material recovery techniques can enhance the availability and quality of raw materials. By bringing together manufacturers, retailers, waste management companies, and policymakers, we can establish shared infrastructure, standardized collection systems, and incentivized recycling programs, optimizing resource utilization, and fostering a circular economy. Various methods, such as isostandardized quantification and microscopy, aid in identifying textile materials. Thermal properties play a crucial role in textile comfort, with factors such as molecular structure and crystallization affecting thermal conductivity. Thermogravimetric analysis (TGA) is used to analyze the thermal stability before pyrolysis. NIR technology coupled with machine learning improves textile waste sorting efficiency. Recent advancements include AI-driven robots, achieving recognition rates of over 95% for sorting textile waste. Near-infrared devices and infrared spectrometers enhance the identification accuracy of textile materials, even distinguishing between synthetic and natural fibers with up to 100% accuracy.

**Figure 3:**
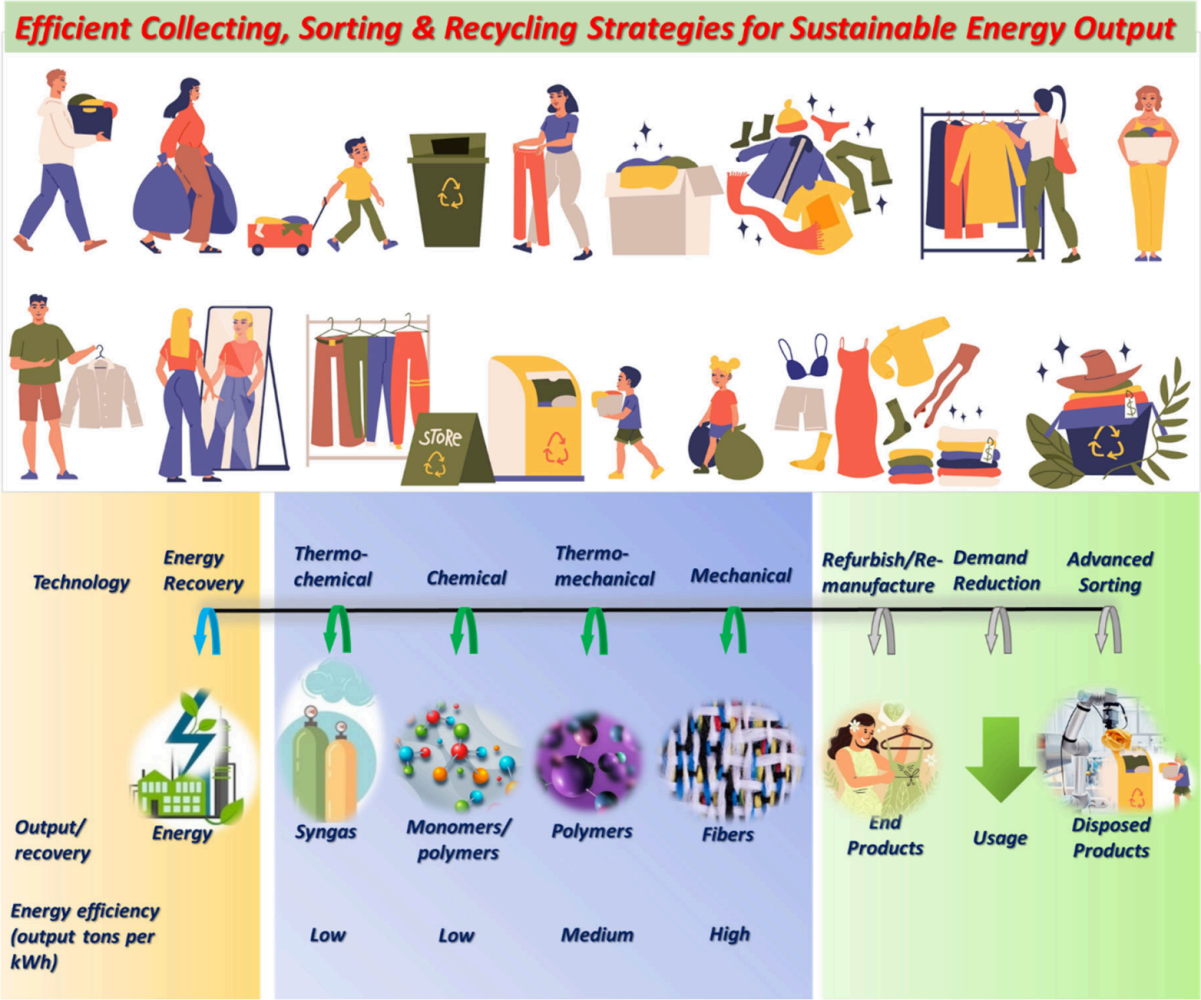
Ingredients for success in collection, sorting and recycling, and technological advancements in recycling processes and their energy output.

Reflectance-FTIR (r-FTIR) spectroscopy provides a non-destructive and non-invasive method for quick textile analysis. Micro-ATR-FTIR spectra helps identify various plastics with high certainty.^33^ Key factors influencing recognition and classification accuracy include considering physical properties like fabric color and yarn thickness, employing transfer learning for computational efficiency, and utilizing fully automated architectures for feature extraction and classification. The challenges confronting textile recycling, including fabric degradation and technological limitations, add complexity to the process, making viable solutions appear distant. Yet, a significant hurdle lies in the scarcity of participants in the collection and sorting sector, impeding progress toward a more sustainable textile industry. Companies like H&M and Levi’s are providing leading innovative garment collection programs that reward customers for recycling old clothes.^37^ H&M has operated a global collection initiative since 2013, allowing customers to drop off clothes from any brand at their stores, with items sorted, resold, repurposed, or recycled, and participants receiving a 15% discount voucher. Similarly, Levi’s, through a partnership of its collaborators, offers a 20% discount for customers who bring in clothes or shoes from any brand. Resale is rapidly gaining popularity, especially among younger consumers, where Levi’s is planning to launch “Levi’s Secondhand”, a resale platform. These efforts aim to reduce textile waste, promote sustainability, and contribute to a circular economy, which other brands are encouraged to adopt. Meanwhile, pre-processing, involving tasks like cleaning and cutting fabrics, remains complex and costly, acting as a bottleneck to scaling textile recycling efforts. Integrating pre-processing into fiber sorting could unlock synergies across recycling methods, but achieving high-quality pre-processing at scale remains challenging. Cutting-edge technologies like Fibersort by Valvan Bailing Systems are also emerging to enhance sorting efficiency, enabling the categorization of textiles based on color, weave type, and fiber content.^36,38^ Despite manual sorting’s economic challenges, it dominates the industry, with resale items fetching higher prices in Western markets than in international second-hand markets. Yet, the resale market remains the primary revenue source, with non-reusable materials expected to increase alongside collection expansion. Non-household textile waste, including post-industrial, preconsumer commercial, and post-consumer commercial textiles, makes up about 15% of Europe’s textile waste. These waste streams pose distinct challenges, from collection to recyclability. Post-industrial waste, originating from manufacturing processes, amounted to 0.5 million tons currently, with a modest growth projection to 0.6 million tons in the near future. Recycling challenges arise from production, fragmentation, and chemical usage in the textile value chain,^39^ where the post-consumer commercial waste, resulting from retail disposal of 0.2 million tons, is expected to slightly increase to 0.3 million tons. Limited collection and sorting complexities hinder recycling, compounded by fragmented collector and sorter landscape. While commercial waste from establishments like hotels totaled around 0.4 million tons, it is projected to rise slightly to 0.5 million tons. Low collection rates, the absence of regulated schemes, and sorting challenges impede recycling efforts, requiring potential policy interventions and increased consolidation, especially due to the dominance of textile rental companies. Educating consumers about the environmental benefits of textile recycling and promoting sustainable consumption habits can stimulate the demand for recycled products, creating a positive feedback loop for the recycling ecosystem. As for the sorting process, revealed that various mechanisms exist for collecting and sorting textile waste, ranging from NGOs and charitable trusts to municipal waste management agencies to reshape the post-consumer behavior even at domestic level (Figure 3). Since, it is imperative to invest in research and development to improve textile-recycling technologies, foster partnerships and collaborations across industry stakeholders to streamline the recycling supply chain, implement consumer education initiatives to raise awareness and demand for recycled textiles, and advocate for supportive policies and regulations to incentivize textile recycling and circular economy practices for sustainable future in fashion industry.

## Technological Advancements in Manufacturing and Recycling Processes

The textile industry’s linear production model has long fueled resource depletion and environmental harm. Cost-effective and high-quality textile recycling is paramount in achieving resource conservation, waste reduction, and sustainable practices. Effective collaboration between different organizations could yield an innovative solution for recycling blended materials into high-quality fabrics and yarns. For example, through a cutting-edge hydrothermal process, the Hong Kong Research Institute of Textiles and Apparel (HKRITA) and the H&M Foundation have perfected the art of fully separating and recycling cotton and polyester blends.^40^ This eco-friendly method harnesses the power of heat, water, and less than 5% biodegradable green chemicals, ensuring minimal environmental impact while maximizing cost efficiency. By licensing this transformative technology widely, HKRITA is poised to revolutionize the textile industry, providing widespread access and making a significant contribution to sustainable material recycling on a global scale. Similarly, Tyton Biosciences developed a water-based hydrothermal solution for recycling garments made from polyester, cotton, and polycotton blends. This innovative process effectively segregates cotton from polyester by disassembling it into monomer components, which are then reassembled into a brand new polyester. Additionally, tree pulp can be replaced with man-made cellulosic fibers (MMCFs), further enhancing sustainability. Recycled polyamide demonstrates lower carbon dioxide emissions compared to virgin polyamide/nylon, with Patagonia incorporating recycled polyamide in over 80% of its apparel. However, the need for specialized handling methods for different materials presents a challenge that could potentially restrain market growth. Future Market Insights’ latest study, “Textile Recycling Market”, provides comprehensive insights into current trends and prospects using a 360° research approach. Key takeaways from the report include North America leading in market share, driven by growing awareness of textile waste’s environmental impact and various public awareness campaigns promoting textile recycling. Europe holds the second-largest market share, with initiatives like the Sustainable Clothing Action Plan in Germany, France, and the UK, and the UK’s Textile 2030 initiative. The Asia-Pacific region is expected to grow rapidly in the textile recycling market, with countries such as South Korea, Japan, and China leading the way. Notable collaborations include Hyosung’s partnership with municipal governments for manufacturing nylon fabrics from recycled fishing nets and Vegan Tiger’s creation of textiles from materials like stickers, gift wrap, and paper. Several key players in competitive textile recycling are advancing the industry through collaborations and technological innovations, such as (mentioned in a recent report by Textile World) Birla Cellulose’s collaboration with Textile Genesis for transparency solutions and Circ’s collaboration with Zara to launch a women’s wear collection made from recycled materials. As the industry progresses, addressing challenges and leveraging opportunities will be crucial in achieving sustainable textile recycling practices.^41^ Various general recycling methods, including textile-to-non-textile, fabric-to-fabric, yarn-to-yarn, fiber-to-fiber, and polymer-to-polymer recycling, have been developed and implemented by companies worldwide, where multiple recycling technology archetypes exhibit differences, particularly in their energy efficiency and their capability to maintain or restore virgin quality, as indicated by a recent report by McKinsey & Company on scaling textile recycling in Europe for turning waste into value (Figure 3). Generally, the desirable traits of returning to virgin quality and energy efficiency are inversely related. This creates a trade-off between recycling textile waste through an energy-effective (and therefore cost-effective) process and one that produces virgin-quality output. Consequently, a long-term solution likely involves a combination of recycling technologies targeting different market segments. Moreover, these technologies could benefit from synergies through collaboration. For instance, the non-spinnable portion of output from mechanical recycling could undergo further recycling via a chemical process. While these technologies offer significant potential, challenges such as low-valued end products and unaffordable costs persist, hindering profitable recycling.

### Chemical and Biochemical Recycling

The quality and value of recycled products often fall short of expectations, particularly in textile-to-non-textile, fabric-to-fabric, and yarn-to-yarn recycling. Issues such as poor quality, style limitations, and fiber damage pose significant challenges. In the quest to make the textile industry more sustainable, the traditional recycling methods, mainly mechanical and chemical processes, though beneficial, have limitations by either degrading textile quality or lacking scalability. These conventional recycling methods struggle to handle the complexity of textile blends and synthetic fibers, impeding the industry’s shift toward sustainability.^42^ Moreover, current methods for dye removal are time-consuming, generate wastewater, and compromise the properties of recycled polymers.^43^ Poly(ethylene terephthalate) (PET) textiles present a significant environmental threat due to their slow degradation and contribution to microfiber pollution, necessitating fundamental changes in the fashion industry’s business model.^25,44,45^ Polyester fabrics made from PET can last for hundreds of years in the environment and release harmful microplastic fibers during washing.^24^ While mechanical recycling of PET degrades fiber quality, limiting its high-value applications, chemical recycling methods like glycolysis, methanolysis, hydrolysis, and enzymatic depolymerization show promise by breaking PET into reusable monomers for new, high-quality fibers.^24^ Glycolysis is particularly noted for its milder, more sustainable conditions.^45^ Advances in catalytic processes could improve recycling efficiency, making it more scalable and supporting sustainable textile production. However, microfiber pollution remains a challenge, requiring innovations in materials to reduce fiber shedding and improve filtration in washing machines. Systemic changes in the fashion industry, including a circular economy focused on recycling, reuse, and sustainable production, are essential. This will require brands to invest in recycling infrastructure and design products that are easier to recycle. Despite the potential of chemical recycling, addressing microfiber pollution and advancing circular economy practices are key ongoing challenges.^46^

The process of hydrolysis in textile recycling, particularly for polyester, breaks down materials into monomers such as terephthalic acid (TPA) and ethylene glycol (EG), which can be reassembled into new polyester. This is especially useful in separating polyester–cotton blends. For natural fibers like cotton, hydrolysis produces glucose, which can be processed into bioethanol or other biochemicals.^47^ The primary uses of hydrolyzed products include repolymerization into new fibers, biochemical conversion into biofuels or organic acids, and serving as chemical feedstocks in industrial processes. These applications contribute to a more circular economy by reducing the reliance on virgin resources. Side products from hydrolysis vary based on materials and process conditions. Common examples include oligomers (shorter chain molecules), acidic byproducts (from acid–based hydrolysis), impurities from dyes or finishes, and cellulosic residues from incomplete hydrolysis of natural fibers. These byproducts may require additional handling to recover valuable components or ensure safe disposal. While, among the proposed solutions, alkaline hydrolysis emerges as a promising method, its sustainability remains under scrutiny. Alkaline hydrolysis has emerged as a promising technology for recycling synthetic fibers, such as polyester and nylon. Using an alkaline solution, typically sodium hydroxide (NaOH), at high temperatures and pressures, this process breaks down polyester textiles into reusable monomers, such as terephthalic acid (TPA) and ethylene glycol (EG), which can be used to produce new fibers. It is particularly effective for polyester–cotton blends as it dissolves the polyester while leaving the cellulose fibers intact. Alkaline hydrolysis is favored for its ability to recover high-purity monomers under mild conditions, contributing to a circular economy in textile production. However, challenges exist in scaling the process due to the need for precise control over the temperature, pressure, and solution concentration, adding complexity. It is less effective for natural fibers, such as cotton and wool, and requires careful management of the alkaline solution to prevent hazardous waste. Improvements in catalyst design and optimization of reaction conditions could enhance the scalability and economic viability of this method.^45^ Additionally, the integration of alkaline hydrolysis with other recycling technologies could help manage a wider range of textile waste more efficiently.

### Mechanical and Thermochemical Recycling

**Figure 4:**
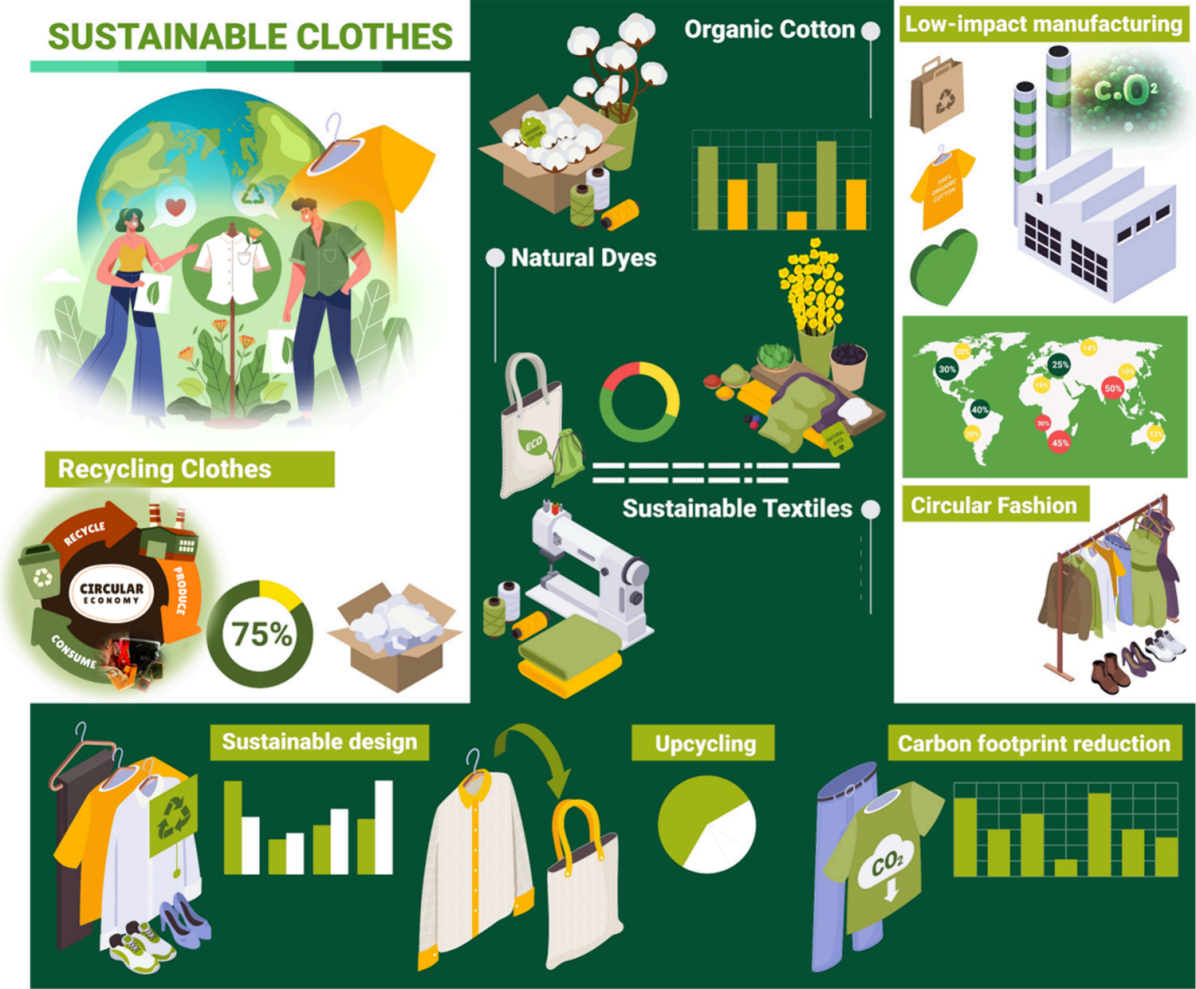
Model for the manufacturing of sustainable clothes and ingredients for the circular economy.

Similarly, recycling wool offers a sustainable solution to reduce the environmental impacts associated with wool production and disposal. Despite its potential, open-loop wool recycling faces challenges such as low-value products and inferior quality.^15^ Closed-loop recycling encounters hurdles related to dye removal and protein dissolution, limiting its feasibility.^14^ Chemical processes presenting closed-loop opportunities in textile recycling into regenerated cellulose fibers show promise in meeting the growing demand for textile fibers, yet their utilization remains limited due to scalability challenges. Despite offering high-value fiber production, it faces obstacles such as solvent toxicity, high energy consumption, managing fiber properties, and identifying different cellulose fibers, thus prompting efforts to enhance solvent reuse and reduce costs.^5,44^ Challenges such as unaffordable costs and environmental concerns persist, hindering widespread adoption of these recycling technologies. In contrast, mechanical recycling stands as the primary method, known for its scalability and lower costs, although it yields materials of reduced quality. Mechanical fiber recycling involves disassembling fabrics through shredding or cutting with established processes for cotton and wool but limited applications for synthetic fibers. Additionally, mechanical processes transform agricultural byproducts into fibers for textile production, offering sustainable alternatives. While mechanical recycling generally consumes less energy compared to virgin fiber production, comprehensive environmental impact assessments are needed. Overall, both methods play crucial roles in textile recycling, each presenting distinct strengths and challenges in sustainability and scalability, while enterprising researchers are tackling the formidable task of recycling cellulosic materials, aiming to alleviate the strain on our planet. Embracing innovative approaches like thermochemical recycling holds promise in repurposing textile waste into valuable resources, offering a ray of hope amid these challenges. Thermochemical processes for textile waste recycling include pyrolysis, gasification, and hydrothermal carbonization, which break down complex polymers into simpler molecules. These methods are effective for handling mixed or contaminated waste that mechanical recycling cannot process. Pyrolysis, for instance, decomposes textiles without oxygen, producing char, bio-oil, and syngas that can be used for energy or refined into chemicals such as benzene and toluene. Gasification partially oxidizes textile waste at high temperatures, generating syngas for electricity, heat, and liquid fuel production. Hydrothermal carbonization heats waste under pressure in water, producing hydrochar, a sustainable fuel or soil amendment alternative to coal. Co-recycling is also gaining attention for its ability to process different waste types together, such as textiles and plastics. For example, co-pyrolysis of polyester textiles and polyethylene plastic produces more liquid fuels than processing them separately. Similarly, the blend of recycled cotton with PET plastic results in stronger fibers for carpets and insulation. Co-gasification of mixed textile and plastic waste is highly effective in waste-to-energy plants, maximizing energy recovery. Chemical recycling can break down polyester textiles and PET plastics together, producing new PET resins for packaging or fiber production. These approaches help manage complex waste streams and convert them into valuable products, reducing environmental impact. As the textile industry progresses toward circularity, integrating different bio-based processes, hydrolysis, and thermochemical recycling methods will be crucial. Policymakers must prioritize regulatory frameworks and incentives to incentivize sustainable practices, whereby embracing innovation and systemic change, industry can pave the way for a more sustainable future (Figure 4). To truly gauge their environmental impact, a comprehensive life cycle assessment is imperative. At the heart of the matter lies the transformation process of recovered cotton into a cellulosic fiber. Integrating cutting-edge technologies such as viscose or lyocell production from recycled cotton holds the key to enhancing recycling efficiency.

### Advancements, Modifications, and Integration of Manufacturing/Upcycling/Recycling Processes

Pretreatment steps using acids, alkalis, or ionic liquids play a crucial role in enhancing the efficiency of hydrolysis, particularly for materials such as PET fibers. Alkali pretreatment, for instance, improves lignin solubilization and reduces cellulose crystallinity, yielding high glucose outputs, while minimizing fermentation inhibitors. Ionic liquid pretreatment, on the other hand, offers environmentally friendly solutions for cellulose dissolution. Meanwhile, acid pretreatments have been explored for their ability to hydrolyze complex structures, albeit with challenges, such as side product formation and equipment requirements. Yet, the potential of acid pretreatments for enhancing biodegradability and cellulose availability remains promising, especially for materials like cotton. Different fermentation techniques, including solid-state fermentation (SSF), separated hydrolysis fermentation (SHF), simultaneous saccharification and fermentation (SSnF), consolidated bioprocessing (CBP), and submerged fermentation (SMF), provide versatile options for ethanol production from textile polymers, each with its distinct advantages.^48^ The urgency of this endeavor becomes apparent when we consider the staggering rise in global textile production over the years. Textiles degrade with use and washing, hampering mechanical recycling efforts. Alkaline degradation of polyester and the Blend Rewind process represent notable strides, but their environmental impact warrants rigorous evaluation through life cycle assessments. However, emerging enzymatic and biological processes offer a promising solution. Thermochemical recycling presents a sustainable alternative by breaking down complex polymers into constituent components, enabling the production of new materials and energy generation. This process offers versatility in handling various textile types and blends, paving the way for closed-loop systems, efficiently utilizing textile waste, reducing landfill burden, and conserves resources. Additionally, it yields high-quality recycled materials or biofuels, contributing to the energy efficiency. Furthermore, it valorizes low-value textile residues and promotes sustainable practices across the supply chain. Bio-based processes for textile recycling encompass various methods, including biological decomposition, enzymatic depolymerization, and fermentation. Biological decomposition involves composting and anaerobic digestion, offering eco-friendly alternatives to incineration and landfill while not being extensively applied to textiles. Enzymatic depolymerization breaks down textile polymers into monomers, with potential applications in recycling various materials. Fermentation utilizes microorganisms to produce useful products from organic substrates, with approaches like solid-state and submerged fermentation showing promise in textile waste utilization, where the enzymatic hydrolysis process employing different fermentation techniques catalyzed by cellulases (SSF, SHF, SSnF, CBP, and SMF) enables the conversion of various textile wastes into valuable products, such as glucose, essential for bioethanol production. Despite advantages such as low energy demand and renewable resources, challenges such as feedstock pretreatment and enzyme costs hinder commercial viability. Nevertheless, the high cellulose content in end-of-life textiles presents opportunities for efficient recycling. Sustainable development of bio-based textile recycling requires addressing economic and environmental considerations, necessitating further research and development to overcome technical and economic challenges. These innovative approaches target specific polymers and fabric types to overcome the complexities of textile materials. Enzymes exhibit specificity, enabling selective breakdown without compromising material integrity, leading to high-quality recycling and the creation of virgin-quality textiles. This biomanufacturing approach utilizing natural dyes and organic raw materials presents a sustainable alternative to traditional methods and fosters innovation for textiles with unique properties. Despite their promise, challenges, such as dye reuse and resource efficiency, must be addressed through further research and development. Novel microorganisms need to be cultivated to enhance substrate conversion rates, while sustainability considerations such as energy consumption and water usage must be carefully managed. Anyhow, different technologies serve different needs depending on the type of textile waste, desired end products, and available resources. Yet, with collaborative effort and interdisciplinary research, these challenges can be surmounted, paving the way for a more sustainable future in the textile industry. In general, recycling and upcycling are both vital methods for managing textile waste but take different approaches to sustainability. Recycling breaks down used textiles into raw materials, such as fibers, which are repurposed into new products. It conserves resources, reduces landfill waste, and operates on a large scale with an established infrastructure. Upcycling, however, transforms discarded textiles into higher value products without degrading material quality, adding value through craftsmanship, and extending the life of garments. Both approaches face challenges, especially with complex material blends such as polyester–cotton, which complicated sorting and preprocessing. Recycling requires precise sorting to maintain raw material quality, while upcycling struggles to find suitable materials for creative reuse. Although recycling is more efficient for large-scale waste management, it can degrade material quality and is energy- and water-intensive. Upcycling, while preserving or enhancing material quality, is labor-intensive, difficult to scale, and economically less viable due to high production costs and inconsistent consumer demand. Recycling benefits from global integration and cost effectiveness, whereas upcycling faces challenges in scale, regulation, and market acceptance. Upcycling also lacks regulatory standards, leading to inconsistent product quality and safety, unlike recycling, which follows established guidelines. While upcycling promotes creativity and sustainability, it requires targeted solutions to address issues of scalability, economic feasibility, and market growth.

The comparison in Table 1 highlights that different technologies serve different needs depending on the type of textile waste, desired end products, and available resources. While mechanical recycling remains the most widely used and cost-effective option, innovations like enzymatic and chemical recycling offer the potential for higher quality output at a greater cost. Upcycling and fiber-to-fiber recycling cater to niche markets with a focus on sustainability and creativity, whereas methods such as pyrolysis and hydrothermal processing provide energy recovery solutions. The right choice depends on balancing the environmental impact, economic feasibility, and product demand.

**Table 1:** Textile Waste Recycling vs Upcycling Technologies

**Technology**	**Target Products**	**Applicable Scale of Operation**	**Relative Costs**	**Advantages**	**Challenges**
					
**Mechanical Recycling**	Fibers, Yarn, Insulation	Small to Large	Low to Moderate	Established, cost-effective	Decreased fiber quality over time
					
**Chemical Recycling**	Monomers, New Fibers (e.g., PET)	Medium to Large	High	High purity, preserves fiber quality	Complex, high energy/water usage
					
**Enzymatic Recycling**	Monomers, Cellulose-based Fibers	Small to Medium	Moderate to High	Selective, eco-friendly	Still in development, scalability issues
					
**Pyrolysis (Thermal)**	Synthetic Oils, Gas, Carbon Black	Medium to Large	High	Converts complex mixtures, energy recovery	High temperature, requires feedstock prep
					
**Hydrothermal Processing**	Fibers, Recycled Polymers	Medium to Large	High	Efficient polymer recovery	High energy and water consumption
**Solvent-based Recycling**	Fibers, Dissolved Polymers	Small to Medium	Moderate	Closed-loop, high-quality fibers	Requires specific solvents, costly
					
**Fiber-to-Fiber Recycling**	New Fibers, Blended Fabrics	Small to Medium	Moderate	Maintains fiber integrity, closed loop	Limited feedstock compatibility
					
**Biodegradation/Composting**	Organic Residues	Small to Large	Low to Moderate	Eco-friendly, reduces landfill waste	Limited to biodegradable fibers
					
**Upcycling (Creative Reuse)**	Fashion, Accessories, Home Goods	Small to Medium (Artisanal)	Low to Moderate	High-value products, creativity involved	Limited scalability, labor-intensive
					
**3D Printing with Recycled Fibers**	Prototypes, Custom Products	Small	High (initial)	Customization, waste minimization	High initial cost, specialized equipment

### Conclusive Remarks and Future Perspectives

The current landscape of textile recycling and upcycling emphasizes the urgent need for transitioning toward a circular economy in the textile and fashion industry, where the linear model of “take-make-dispose” has significantly contributed to environmental challenges, including massive textile waste generation and CO_2_ emissions. Despite advancements in recycling technologies, issues such as inadequate waste collection, sorting inefficiencies, and limited scalability of technologies continue to hinder the efficient management of textile waste. Enzymatic depolymerization and thermochemical upcycling hold great potential in breaking down complex polymers, such as polyester and blended fabrics. These technologies provide opportunities for recovering high-purity monomers, which can be reused to manufacture new fibers, contributing to a closed-loop recycling system. Innovations like co-pyrolysis and co-gasification offer opportunities for recycling mixed textile waste and plastic waste together. These methods improve resource recovery and can produce higher value products, such as biofuels, syngas, and chemical feedstocks, allowing for efficient management of blended or contaminated textile waste. The EU’s upcoming mandates on textile waste collection by 2025 and the broader push for circular economy policies create opportunities for scaling textile recycling efforts. Regulations encouraging the separation of textile waste and the prohibition of unsorted waste exports could streamline the sourcing of recyclable feedstock. Automated sorting technologies such as near-infrared scanning systems (NIRS) and AI-driven robots offer immense potential for improving the efficiency of textile sorting. Innovations in pre-processing, such as cleaning and fiber separation, can enhance the quality of recovered materials and reduce contamination in recycling streams. Upcycling technologies present a transformative opportunity to convert low-value textile waste into high-quality products, creating new economic avenues. Emerging innovations such as blending recycled fibers with new materials or enhancing material properties (e.g., strength) in upcycled fibers present significant opportunities for value addition.

Future research should focus on optimizing chemical recycling processes such as glycolysis, methanolysis, and enzymatic depolymerization for scalability. Key areas include improving catalytic efficiency, reducing the environmental footprint of solvents, and finding cost-effective ways to manage by-products. The future of textile recycling depends heavily on improving waste collection and sorting. Investing in technologies such as automated sorting systems, robotic handlers, and AI-based identification of fiber blends can dramatically enhance the efficiency and output quality of recycled materials. Governments and industry leaders should collaborate to create policies that incentivize recycling and penalize the use of unsustainable practices. Extended producer responsibility (EPR) schemes, eco-design requirements, and tax incentives for using recycled materials can drive the adoption of circular business models across the fashion industry. Addressing microfiber pollution caused by synthetic fibers is critical. Future research should explore material innovations that reduce fiber shedding during washing and promote filtration technologies for washing machines. In addition, life cycle assessments (LCAs) of recycling processes are essential for minimizing environmental impacts. Encouraging sustainable consumption habits and educating consumers about textile recycling can boost participation in recycling programs. Brands should invest in awareness campaigns and collaborate with NGOs and governments to promote the benefits of recycling and upcycling. Collaboration among manufacturers, retailers, recyclers, and policymakers is essential to developing shared infrastructure for textile waste management. Joint efforts in establishing standardized collection systems, recycling technologies, and market mechanisms for recycled products are key to overcoming fragmentation in the supply chain.

To meet the challenges in textile waste recycling and upcycling, future approaches should prioritize innovation in chemical recycling, regulatory enforcement, and advanced sorting technologies. Addressing the remaining technological, economic, and consumer behavior challenges will enable the transition to a fully circular textile economy, minimizing environmental impacts and creating economic opportunities.
